# CASCADE: a community-engaged action model for generating rapid, patient-engaged decisions in clinical research

**DOI:** 10.1186/s12874-025-02565-7

**Published:** 2025-07-01

**Authors:** Bridgette L. Kelleher

**Affiliations:** Department of Psychological Sciences, Purdue University, 703 3rd Street, West Lafayette, IN 47906 USA

**Keywords:** Community-based participatory research, Decision making, Clinical trials, Patient engagement, CASCADE, Delphi panel, Project wellcast, Patient acceptability

## Abstract

**Background:**

Integrating patient and community input is essential to the relevance and impact of patient-focused research. However, specific techniques for generating patient and community-informed research decisions remain limited. This manuscript describes a novel CASCADE method (Community-Engaged Approach for Scientific Collaborations and Decisions) that was developed and implemented to make actionable, patient-centered research decisions during a federally funded clinical trial.

**Methods:**

The CASCADE method was developed to facilitate decision-making, combining techniques from a variety of past methodologies with new approaches that aligned with project constraints and goals. The final result was a series of procedures that spanned seven thematic pillars (1) identifying a shared, specific, and actionable goal; (2) centering community input; (3) integrating both pre-registered statistical analyses and exploratory “quests”; (4) fixed-pace scheduling, supported by technology; (5) minimizing opportunities for cognitive biases typical to group decision making; (6) centering diversity experiences and perspectives, including those of individual patients; (7) making decisions that are community-relevant, rigorous, and feasible. The final approach was piloted within an active clinical trial, with the primary goal of describing feasibility (participation, discussion topics, timing, quantity of outputs).

**Results:**

The inaugural CASCADE panel aimed to identify ways to improve an algorithm for matching patients to specific types of telehealth programs within an active, federally funded clinical trial. The panel was attended by 27 participants, including 5 community interest-holders. Data reviewed to generate hypotheses and make decisions included (1) pre-registered statistical analyses, (2) results of 12 “quests” that were launched during the panel to answer specific panelist questions via exploratory analyses or literature review, (3) qualitative and quantitative patient input, and (4) team member input, including by staff who represented the focal patient population for the clinical trial. CASCADE pillars were successfully integrated to generate 18 initial and 6 final hypotheses, which were translated to 19 decisional changes.

**Conclusions:**

The CASCADE approach was an effective tool for rapidly, efficiently making patient-centered decisions during an ongoing, federally funded clinical trial. Opportunities for further development will include exploring best-practice structural procedures, enhancing greater opportunities for pre-panel input by community interest-holders, and determining how to best standardize CASCADE outputs.

**Trial registration:**

The CASCADE procedure was developed in the context of NCT05999448.

## Background

Integrating patient and community input into decision making is essential to the relevance and impact of patient-focused science [1]. However, specific techniques for community-informed decision making remain limited. Practical, in vivo community engagement techniques are particularly lacking, with most guidelines focusing on the broad approaches to community-engaged research rather than the specific technical strategies that researchers can use to involve patients and communities in real-time. The present manuscript describes a novel CASCADE method (Community-Engaged Approach for Scientific Collaborations and Decisions) recently developed and implemented to make actionable, patient-centered research decisions in the context of a federally funded clinical trial. This manuscript first describes the justification and empirical motivation for developing CASCADE, including how the approach differs from other community-centered and consensus-generating methods. Next, the technical protocol for implementing CASCADE is described as implemented during an active clinical trial. Finally, key takeaways and next steps for methodological development and validation are discussed.

### Methods for summarizing consensus across patients and community-members

The voice of the patient is central to any clinical research endeavor. Patient engagement in research has been systematically defined as *“the active*,* meaningful*,* and collaborative interaction between patients and researchers across all stages of the research process*,* where research decision making is guided by patients’ contributions as partners*,* recognizing their specific experiences*,* values*,* and expertise.”* [3, p. 682]. A variety of methods have been used to engage patients in healthcare and research contexts [3], including involvement of a patient advisory councils [4], patient-led provider training [5], and co-designing research programs [6]; large-scale meta-analyses have supported the efficacy of such programs on health outcomes, particularly when communities are directly involved in health-related interventions [7]. More passive methods for considering patient experiences are also common, such as the evaluation of patient behavior (e.g. attrition, compliance) or patient-reported surveys to assess acceptability of healthcare interventions [8]. Increasingly, patient communities are self-organizing to impact and control research decisions, including by developing research resources such as registries [9, 10] and, in some cases, directly financing and co-creating research relevant to their community [11].

Patient-engaged research can be conceptualized as a type of participatory research, which broadly aims to engage potential users of research into the design and application of the research itself [1]. Participatory methods, including community-based participation research (CBPR) methods, have historical roots in Kurt Lewin’s *action research* movement, which aimed to engage minority participants in the translation of complex social issues to social action through a sequence of fact finding, taking action, and evaluating impact [12]. At present, CBPR is generally characterized as a collaborative research approach that integrates equitable input from community, organizational, and research interest-holders [13, 14]. Israel and colleagues [13] have summarized key tenents of CBPR, including many principles relevant to patient in research-related decisions. However, the current status quo is that few patient-focused endeavors fully align with these CBPR tenents. One particular challenge to CBPR is the often-unclear process for how to best synthesize patient perspectives into actionable outputs [13]. Rigorous qualitative methods that are often used in CBPR, such as focus groups and intensive interviews [15, 16], are also often time-consuming and resource-intensive, posing challenges for rapid decision-making contexts. Methods for more generally engaging with community advisory groups are not well-standardized, and there is little accountability for researchers to integrate and act on community input in these contexts. Thus, additional frameworks are needed to translate CBPR into acute, patient-engaged decision-making contexts.

### Methods for building consensus across experts and lay experts

A variety of methods have been developed to generate consensus or agreement in medical research [17, 18] and offer a starting point for building a model for how to generally build consensus on patient-relevant topics. For example, the Delphi method [19] is a highly popular, systematic process for making complex decisions by iteratively integrating expert input toward consensus across multiple rounds of anonymous expert feedback. However, in contrast to CBPR principles, this structure assumes that group-based decisions provide greater value and stability than individual input [20], and that discussion weakens decision-making by introducing biases and uneven input [19–21]. Other models for consensus and decision-making have – such as the RAND/UCLA Appropriateness Method [22] and consensus development conferences [23] - typically include more discussion and input from lay experts [17, 23]. However, similar to Delphi panels, these methods generally center expert opinion and require extensive resources to execute, limiting utility for CBPR.

A fourth common model for consensus - nominal group technique [24, 25] – incorporates several elements that more closely align with the goals of CBPR. Similar to other consensus models, nominal group technique involves a multi-step phase that includes structured presentation of input, feedback to the group, discussion, and voting to rank-order outputs. A key distinction of this method is that prior to this process, group members engage in is “nominal” activities such as independent, written responses to pre-determined prompts, with the goal of minimizing the biases and power imbalances and enhancing creative outputs [25]. Technique developers Van de Ven and Delbeco (1971) explicitly note that providing time for individual reflection and input prior to group discussion may “encourage the generation of minority opinions and ideas” and “alleviate… covert political group dynamics which are difficult to develop when writing,” aligning with CBPR principles. Although nominal group techniques are typically applied to gather consensus among experts, the approach is increasingly used to identify consensus amongst patients [26, 27], providing a potential starting point for integrating patients in more rapid decision-making contexts.

A common criticism of consensus-driven methods, including nominal group technique, is the potential to dilute novel ideas and focus policy and decisions at the level of a “lowest common denominator” [23]. Indeed, a variety of cognitive biases impact decision making, particularly in group contexts, and are purported to impact patient outcomes [28]. Leveraging voting to determine outcomes – as is typically the case in nominal group methods – may also reinforce power dynamics by biasing outcomes to majority opinion. To minimize the potential impact of such biases in consensus generation, Bhandari and colleagues [29] generated a guide to identifying and reducing specific cognitive biases that can compromise group-based decision making. For example, they suggest that iterative rounds of discussion with descriptive feedback and minimize potential the *false consensus effect* [30], a tendency to over-estimate the degree to which others agree with one’s own opinion. Their guidelines provide a useful metric for considering how methodological decisions impact the rigor of consensus-based decisions, particularly when designing new approaches to integrating patient voices into consensus-based research. However to date, no clear protocol for integrating these approaches into research-based decision making has been established. Developing and piloting a protocol is an important first step toward optimizing community-centered decision-making procedures in the context of clinical research studies.

## The present study

Although a variety of CBPR and consensus-based decision-making approaches have been developed, the field lacks a specific, generalizable approach for *applying* these tenents to the more specific scenario of making decisions during a research study. A variety of situations arise during active clinical research that might benefit from patient input. For example, investigators may want to better understand how to improve accessibility of trial procedures, decide how to interpret and act upon unexpected findings, or select new approaches or treatments to pilot. Often, these decisions must be made quickly, precluding the use of time-consuming but rigorous methods such as Delphi panels or focus groups. As a result, investigators may either fail to consider patient input or rely on less standardized approaches, such as feedback surveys or informal advisory boards. New, standardized methods are needed to integrate community-interest holder input into real-time clinical research decision-making, while minimizing biases and logistical challenges inherent to this work.

Addressing this gap became necessary during Project WellCAST, a NIH-funded clinical trial that aimed to identify personalized health approaches for supporting well-being for rare disorder caregivers. Within the trial design, a series of panels were planned to review project data and make changes to the each panel included researchers, biostatisticians, clinicians, and community members. This team was tasked to collaboratively review project data and make decisions about changes to a precision health algorithm that was used to assign patients to treatments. However, no standardized protocol existed for making collaborative, community-informed decisions in such an acute timeframe. As such, an opportunity arose to develop a new, standardized decision-making method that could meet the acute needs of the trial and serve the broader field. The present manuscript introduces the resulting CASCADE method (Community-Engaged Approach for Scientific Collaborations and Decisions), a series of techniques embedded in the decision-making panel to maximize rigor, reproducibility, and community engagement. This manuscript describes the guiding principles, technical steps, and initial outcomes of the CASCADE panel approach, concluding with “lessons learned” and suggested next steps for the field.

## Methods

The purpose of the CASCADE method is to rapidly synthesize multiple sources of data with community and scientific input to make acute research decisions. CASCADE was developed out of necessity, as no step-by-step, standardized model had been proposed that met the following criteria: (1) could be implemented rapidly, (2) maintained accountability to community input, (3) accounted for potential power differentials and cognitive biases relevant to groups, and (4) included standardized decision making guidelines. For example, CBPR-aligned methods such as focus groups integrate community input (#2), are typically set up to minimize biases (#3), and are standardized (#4); however, these methods are highly time consuming to conduct, code, and translate to thematic outputs, which then require interpretation and application into a research context (violating #1). Nominal group techniques provide an appealing starting point for making community-engaged decisions – as nominal techniques can be quickly implemented (#1) with community members (#2) and minimize some group-related biases (#3), however the decisional process typical involves an iterative voting procedure that is vulnerable to majority input and does not align with any particular pre-set criteria for acceptable outcomes (violating #3 and #4).

### Guiding pillars of CASCADE

**Fig. 1:**
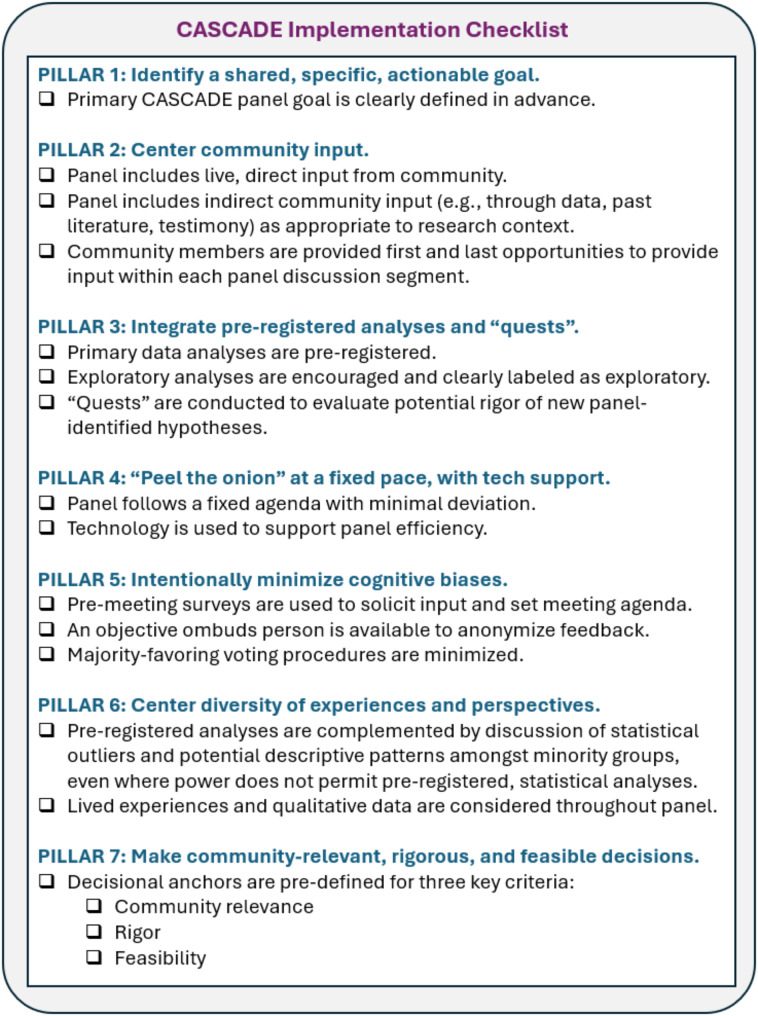
CASCADE Panel implementation checklist

The CASCADE approach was developed practically, with an acute goal of designing a scientifically rigorous process informed by the past literature on CBPR, models for systematic decision making, and science on group-related biases. Efforts were made to identify key defining principles of the decision-making approach that would both satisfy current project needs and could be flexibly applied across a variety of contexts. Core guiding principles are detailed in Fig. 1 with an accompanying implementation checklist. The decision of which pillars to include was largely functional and needs-driven, while also pulling from successful past models that have been described previously and are referenced below. Although the ways in which pillars are fully implemented will vary across study designs, it is expected that these key guiding principles summarize foundational elements that define the CASCADE approach.

#### Pillar #1: Identify a shared, specific, actionable goal

A CASCADE panel functions to answer a specific, pre-defined question. In this way, CASCADE has structural similarities with initial action research approaches that aimed to distil complex issues into actionable progress [12, 31]. In cases in which a clear goal is not fixed in advance, a variety of CBPR principles and techniques can be used to facilitate shared decision making around research questions and agendas [13, 16, 32, 33]. Similarly, a number of methods have been developed to ensure goals are well-described. For example, SMART goals are created to be *s*pecific, *m*easurable, *a*chievable, *r*ealistic, and *t*ime-based [34]. By anchoring to a specific goal, CASCADE promotes action-oriented outputs that directly address a specific project need.

#### Pillar #2: Center community input

Centering community input is a hallmark and central principle of the CASCADE approach and should be accomplished both directly and indirectly. Although methods for integrating community input will vary by project, inaugural panel methods are described here as examples. In the present study, *Direct Community Input* was represented by “peer coaches,” caregivers of children and adults with rare disorders (the target population for the trial) who were paid part-time staff on the project. Within the broader project, peer coaches help design and plan elements of the project, implement a portion of support programs, support recruitment and community engagement, and assist with data interpretation and dissemination. Given we were discussing confidential information, having paid, human subjects-certified staff on our team who could provide input and hands-on perspective was central to the success of CASCADE. Because peer coaches interacted directly with participants about their experiences in the trial, they were also able to offer anecdotal information about their observations and perceptions about patient experiences. They were compensated for participating, similar to all other project staff, promoting equity. *Indirect Participant Input* was represented through both patient-reported and behavioral data, per general field standards [8]. For our specific study, patient-reported data included quantitative survey responses and qualitative responses to open-ended questions. We also indirectly evaluated implied patient experiences by integrating observational proxies of participant outcomes such as drop-out, session completion, and homework completion [8]. We anticipate that as CASCADE panels are conducted by other groups, the specific nature in which community input will be represented will vary widely; however, directly and directly incorporating community interest-holders should be considered a key component of this approach, as detailed in Fig. 1.

#### Pillar #3: Integrate both pre-registered statistical analysis and exploratory “quests”

Centering data in decision making is also important to the CASCADE approach. A primary focus of CASCADE was on pre-registered statistical analyses. The benefits of statistical pre-registration have been well described in the literature [35], building on a rich history of protocol registration that is common, and often required, for medical and clinical research [36]. Pre-registration is important to reducing potential biases, increasing transparency, and minimizing what has been described as “researcher degrees of freedom,” [37] subtle ways in which researchers’ design and analysis decisions can intentionally or unintentionally bias results [35]. In the context of CASCADE, preregistration is particularly important to distinguish planned analyses, which we implemented to test or core hypotheses, from exploratory analyses that functioned to help generate hypotheses for the next wave of data collection.

A second major focus of CASCADE was to develop and evaluate novel hypotheses as the panel unfolded. Here, we incorporated the idea of “Quests,” defined as rapid, targeted data analysis or literature review executed with the purpose of evaluating the relative strength of a new hypothesis. These exploratory analyses were not designed to produce generalizable knowledge about the target population, but rather to consider the strengths and weaknesses of proposed hypotheses and action items. Quests were designed to be limited in scope, capable of being completed in 1 hour or less in between panel meetings, and directly related to specific hypotheses. Quests were completed by project staff, including biostatisticians and student or postdoctoral trainees, and were verified for accuracy after the panel, prior to final implementation. The specific types of data evaluation needed within future CASCADE panels will likely vary by panel purpose. However, including both pre-registered analyses and more flexible, exploratory quests provided a helpful framework for addressing questions raised during the panel, prior to making decisions. As such, both pre-registered analyses and quests should be considered core elements of CASCADE panel approaches.

#### Pillar #4: “Peel the onion” at a fixed pace, with support from technology

The CASCADE model is focused on efficient decision-making, which comes at an obvious and expected cost to discussion depth. We conceptualized our task during CASCADE as peeling an onion, with the understanding that we could only get to so many layers in a given period of time. As such, the agenda for each day was fixed in advance, with minimal deviation, and it was acknowledged that we would not be able to fully explore all possibilities during the project. To maintain this pace, we prepared many of the core documents ahead of the meeting, including statistical analyses, and leveraged pre-meeting surveys to solicit panelist input in advance [24].

**Fig. 2:**
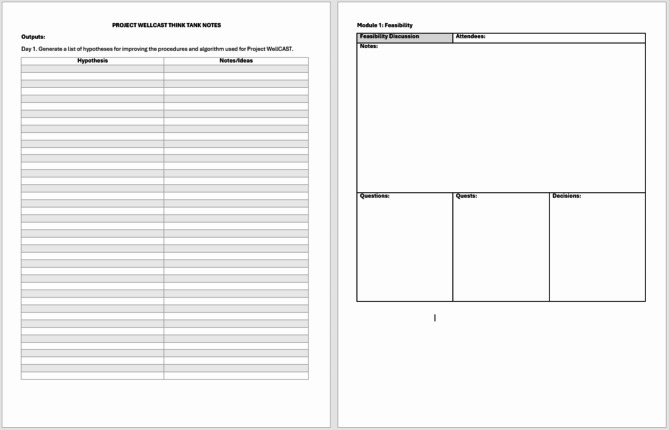
Sample shared note-taking document used to document CASCADE in real time

To maintain a rapid pace, we selectively leveraged technology to support both clerical and data synthesis tasks. Although it is expected that future groups will vary in how technology is implemented in their panels, examples are provided to illustrate how CASCADE was implemented in this specific study. Clerically, we relied on a shared note-taking document on Google Docs [38], accessible to all panelists, that documented (1) key hypotheses generated during the meeting, (2) details of each segment of discussion, along with questions, planned quests (Pillar #3), and decisions, and (3) documentation of all project decisions, including how we satisfied our core decisional criteria (Pillar #7); the shell for this document is displayed in Fig. 2. We also leveraged Zoom’s “record meeting” function to save record of the meeting, used for later verification of discussion, and used the chat feature to supplement live dialogue during the meeting.

To support data synthesis, we also used ChatGPT [39] to summarize – but not thematically analyze – both participant and panelist input. ChatGPT has been previously validated to accurately extract concrete and descriptive themes from qualitative data, however its capacity to conduct thematic analyses and detect nuanced patterns is more limited [40]. Within CASCADE, we used ChatGPT with these constraints in mind by (1) requesting item-by-item synthesis, anchored to a very specific item prompt, (2) never uploading sensitive, personal, clinical, or identifiable data, and (3) cross-validating ChatGPT with other analysis methods, particularly when summarizing participant input (Pillar #2); details of the results of these analyses are beyond the scope of this CASCADE-focused publication, as AI use is not a required element of the CASCDADE process.

#### Pillar #5: Intentionally minimize opportunities for cognitive biases

Consistent with recommendations by Bhandari and colleagues [29], a key goal of CASCADE panel structure is to minimize the impact of cognitive biases on decision-making. Per nominal group technique [24, 25], pre-meeting surveys were used to help panelists engage in creative brainstorming prior to the meeting; having participants describe and justify their ideas in advance was intended to reduce potential for groupthink and facilitator biases [29]. We also included an ombuds procedure whereby participants could communicate via a non-research administrator through a direct, private chat. Given the differences in power and perspectives across groups, with more researchers than community interest-holders in attendance, we did not use majority voting procedures to progress ideas forward like many past consensus approaches, although we did ask participants to nominate their top ideas for discussion via pre-meeting surveys and ensured these were discussed each day. We also started each discussion segment with input from peer coaches and/or reflections on participants’ perspectives to center the discussion on community members’ priorities and give community interest-holders power over the meeting contents. Specific techniques for minimizing biases are expected to vary by research and community context; however, it is recommend that CASCADE panels include key components of pre-meeting surveys, ombuds, non-majority decision making procedures, and starting and stopping each discussion with community voices (Pillar 2).

#### Pillar #6: Center diversity of experiences and perspectives, including for “n = 1” experiences

The CASCADE approach acknowledges that often, clinical decision-making must consider how research will impact participants in the minority, whether defined by demographic or experiential factors. Thus, in addition to adequately powered, pre-registered analyses, we encouraged open discussion of “n = 1” issues, such as challenges that may differentially impact specific subgroups of participants. Where robust statistical approaches are not possible, these discussions are anchored with other sources of data – including past research, lived experiences shared by participants or community representatives, and qualitative findings. Decisions made related to minority opinions were subject to the same standards as other types of data-based decisions (Pillar 7).

#### Pillar #7: Make decisions that are community-relevant, rigorous, and feasible

A major barrier to the implementation of CBPR in research decision-making, as defined by Israel and colleagues [13], is the challenges inherent to synthesizing rich, multifaceted patient data into actionable outputs. A key contribution of the CASCADE approach is formalization of criteria that can be used to make rapid, data-based decisions. Prior to translating a hypothesis into action, our panel required that (1) the action be supported by community, as expressed by peer coaches and/or data collected from participants, (2) the action be supported by at least 2 of the following data sources: quantitative data, qualitative data, past literature, lived experience, (3) the action be feasible within the temporal and financial constraints of the project. Actions that did not meet these criteria were flagged for follow-up outside of the CASCADE panel context, such as to conduct pilot projects or address feasibility barriers through longer-term projects and grants. The panel limited discussion of such endeavors to maximize panel efficiency. Future CASCADE panels should similarly anchor decisions to pre-set criteria for community relevance, rigor, and feasibility, although the specific thresholds for these criteria may vary by study.

### Procedures

This section details key chronological sequence of tasks required to plan and execute the CASCADE approach, including how each step was applied within our project-specific CASCADE panel.

Prior to the CASCADE meeting, we articulated our primary goal that was “fixed” within our grant protocol: “Identify how to improve the Project WellCAST algorithm to better match caregivers to feasible, acceptable, and effective supports.” Next, we pre-registered the specific statistical analyses that would be used to guide our panel discussion and report on the project’s OSF.io site, complementing our prior trial registrations. In the context of our panel goal, analyses focused on how the feasibility, acceptability, and efficacy of the clinical trial treatments varied according to participant and treatment characteristics.

Next, we defined our panel structure. We invited all project co-investigators, research staff (including community interest-holders), research assistant trainees, clinical supervisors, clinician trainees, and biostatisticians. Panelists were encouraged to attend as much of the meeting as possible, with the understanding that other commitments may impact attendance; agendas were adjusted to maximize discussion that was relevant to participants with intermittent availability. To maximize participation, we implemented a hybrid model that included in person and remote options for attendance.

Panelists received a pre-meeting survey that requested specific inputs relevant to the planned discussion, with questions designed to parallel key decision points. Prior to the meeting, panelists received several digital items; a subset of documents were also mailed to remote participants. These items included: (1) a preliminary report of findings, with a focus on descriptive data that are used for pre-registered analyses, (2) agenda and slide deck, (3) links to supplemental descriptions of all measures, procedures, and de-identified data for additional use if needed, (4) hand-written thank you note and project “swag” (sticker, pen) to promote a sense of community and belongingness. Digital documents were provided in a secure, password protected cloud folder.

## Results

The Project WellCAST CASCADE panel occurred July 2024 and facilitated by the project PI (BK) who had prior experience leading interdisciplinary groups toward consensus decisions, including through formalized training in agile leadership for moving groups toward action [41]; the leader was not a member of the focal participant community (rare disorder caregivers) but experienced some shared identity as a parent of children with disabilities.

### Trial context

The panel occurred in the context of needing to make acute decisions about how participants would be matched to a variety of support programs.

### Attendees

Including the facilitator, 27 team members attended across days, including 5 staff who were also community interest-holders, 9 doctoral-level clinical researchers (5 licensed), 3 biostatisticians (two doctoral-level, one masters-level), 8 psychology and special education trainees (2 postdoctoral scholars, 4 graduate students, 2 undergraduate students), 1 research project manager and 1 administrative support staff. Researchers represented 5 institutions across two countries (United States and New Zealand), and community interest-holders represented 4 patient communities.

### Panel structure

**Fig. 3:**
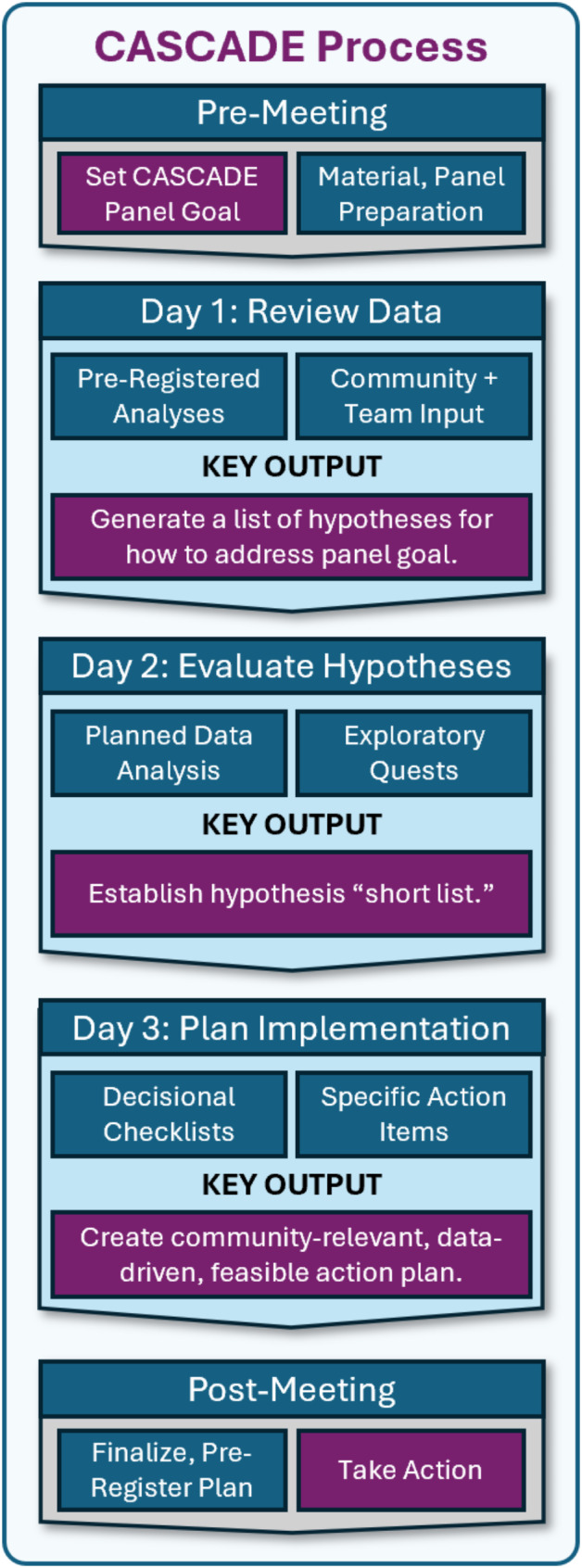
CASCADE three-day itinerary and key outputs

**Fig. 4:**
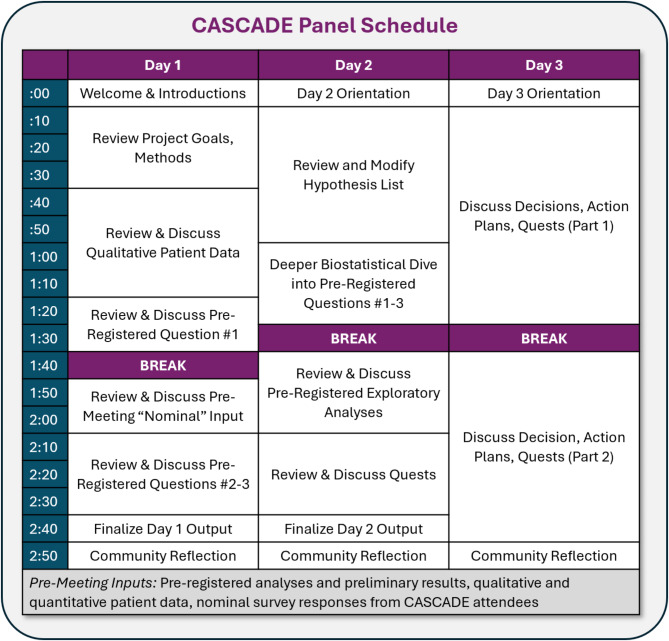
Observed CASCADE Schedule for Project WellCAST Panel (July 2024)

The CASCADE Panel occurred across three consecutive days (Figs. 3 and 4), with sessions held between 2-5PM EST each day to account for multiple time zones of participants, who attended from across the United States and New Zealand. The meeting was administratively supported by an on-site research operations administrator who served as an ombudsperson via secure, private chat and could relay anonymous information to the project team in real time. All attendees were encouraged to update meeting notes in real time via shared documents and were given access to additional technical materials (“Meeting Inputs”) that they could access from personal computers.

### Panel schedule and outputs

Figure 4 details the panel itinerary. Day 1 primarily focused on reviewing project data and generating hypotheses; the end-product was a list of hypotheses about how changes to the algorithm may improve participant outcomes. Day 2 primarily focused on “Quests” through which we conducted exploratory analysis of past WellCAST data and reviewed past literature to estimate the feasibility and impact of various hypothesized improvements; the end-product was a list of final algorithm improvement suggestions. Day 3 focused on establishing a plan for implementing algorithm changes; the end-product was a draft of planned changes that would finalized in the post-panel period by core project staff.

### Hypotheses and quests

Across days, 18 specific hypotheses were suggested for consideration, and 12 quests were undertaken to contextualize the relative strengths and weaknesses of these hypotheses. Quests included specific pilot analyses (*n* = 7; e.g., detailed summaries of why participants dropped out of the study, statistical analyses exploring the degree to which emotional dysregulation related to drop-out), administrative record review (*n* = 1; e.g., clarifying types of employment in demographic data), and reviews of the literature (*n* = 5; e.g., surveying the literature for examples of how personality might be related to group treatment dynamics).

### Decisional outputs

Of the 18 hypotheses initially suggested for consideration, 6 were candidates for immediate action (feasible) and were discussed for relevance to the community and support from past data. An additional 12 hypotheses were earmarked for later follow-up; for example, although there was enthusiasm to consider how the construct of hope may relate to outcomes, a measure of hope was not in the original dataset, and follow-up discussion was planned to consider adding such a measure. Final justification for each CASCADE-generated decision will be published alongside study findings; however, in total, 19 decisional changes were selected that aligned with our criteria for community-relevance, empirically supported, and feasible (Pillar #7). Verbal consent from all panelists in attendance was used to determine final consensus.

### Post-panel action

Final edits to the report and the proposed, preregistered algorithm were sent to the CASCADE team for verification and integration into the next round of project routing decisions, which occurred 5 weeks following the CASCADE panel. Following the meeting, project staff re-reviewed all recordings to check for completeness of documentation and verifying all proposed changes met decisional criteria. Given our goal was highly technical in nature (changing an algorithm), there were also several follow-up steps of identifying specific thresholds, updating code, and piloting and debugging updates. Across stages, these technical changes were constrained to the general scope of decisions made during the CASCADE panel, and the final list of changes, including any technical details that were not explicitly discussed during the panel, were sent to all panelists for verification prior to implementation.

## Discussion

Although a variety of decision-making procedures have been developed for medical contexts, existing procedures typically require substantial time and resources and offer minimum opportunity for patient and community input. This manuscript introduces a new decision-making model, CASCADE (Community-Engaged Approach for Scientific Collaborations and Decisions), designed to systematically integrate scientific and interest-holder inputs to make clinical research decisions. Results from an inaugural CASCADE panel indicated that the methodology facilitated efficient, data-based decision making by a highly interdisciplinary team, with substantial input from community interest-holders.

A primary takeaway from the CASCADE pilot was that decisions could be made with high efficiency using the CASCADE approach. Specifically, in less than 9 hours of panel meetings across three consecutive days, our team was able to efficiently review data, generate hypotheses, consider the relative strengths and weaknesses of these hypotheses, and make an actionable list of decisions. This efficiency was facilitated by several aspects of our CASCADE approach. First, consistent with the nominal group technique for decision-making [24, 25], a variety of inputs were prepared in advance, including a survey to solicit panelist input. In addition to supporting meeting efficiency, soliciting written input in advance was anticipated to minimize potential cognitive biases [29], consistent with many other consensus-generating models [17, 18]. We also leveraged technology to enhance meeting efficiency, including by using AI to rapidly summarize de-identified, non-sensitive meeting inputs. It is important to note that AI is not an inherent element of the CASCADE process, and each research team is expected to make decisions about how to best leverage technology to facilitate rapid data reviews and decision-making during CASCADE panel procedures. As technology continues to evolve, it will be important to continuously evaluate how to harness the power of technology-driven communication platforms, teleconferencing, and AI tools, while also protecting the quality of data and confidentiality of project participants. The CASCADE approach is not yoked to any specific technology use or integration; however, future users are encouraged to carefully consider and harness tech tools that are appropriate to the goals and needs of their projects.

A second key takeaway was the high impact of patient community input on panel decisions, which again reflected a variety of intentional strategies in the CASCADE model design. We integrated patient community input directly and indirectly, including by centering input from paid community representatives who were members of the project staff. These team members were highly knowledgeable about project procedures, engaged directly with patients as part of the trial, and could provide highly specific input informed by both their lived experience and project experiences. Anecdotally, many non-community panelists noted that, consistent with the many benefits of CBPR [14], the research team would have likely interpreted results of analyses differently if not for the interest of these team members, who often provided context and nuance that was not possible to detect from numeric data alone. Structurally, we also observed positive outcomes of starting each discussion period with space for community interest-holders to speak first, which ensured that the “seed” for each discussion was centered on the community priorities and needs. Given the compact schedule for our CASCADE panel, starting with community input was critical to maximizing the impact of community-interest holders on project decisions. Although future adapters of CASCADE may choose a variety of strategies for integrating community members into CASCADE panels, we recommend direct involvement and careful consideration of group dynamics and power differentials (e.g. through pre-meeting surveys, starting and ending segments with community input, evaluating outcomes against community priorities) to maximize the effectiveness of the CASCADE approach.

Several considerations will motivate future phases of CASCADE model development. First, we will consider who should facilitate CASCADE panels. Here, CASCADE was facilitated by the project PI and developer of the CASCADE model, who had prior formal training in group-based consensus generating procedures. To minimize potential for facilitator-related biases, panel procedures were pre-registered, community interest-holders were called upon first to provide input during each segment, and all decisions were made via consensus. However, these procedures do not fully ameliorate the potential for facilitator bias, and future work should explore potential benefits of objective, external facilitators. Second, community input similarly originated from within the project; this decision reflected the need to protect patient and algorithm information during an ongoing trial. However, future projects could explore creative and secure ways to gather broader community input – such as by preparing a separate pre-panel meeting to discuss broad project questions with patient community representatives and relevant foundations – to improve the scope of community input. Such considerations must be made with ethical constraints in mind, and conducted within the scope of ethical review guidelines and responsibilities.

We are also considering several aspects of CASCADE panel structure. First, the specific decision to execute CASCADE across three part-day meetings was somewhat arbitrary, reflecting the estimated minimum time needed to execute activities and the temporal constraints of the clinical trial. Future experimentation could alternate schedules, including those that allow more time to re-solicit panelist input [23] and complete additional quests. Second, we made decisions via consensus, without anonymous voting procedures that are common in decision making models, in part because our community representatives were the minority of panelists. Our panel functioned highly collaboratively and congenially, with no overt conflict across group members or engagement of the ombudsperson that would suggest potential undetected disagreement. Nonetheless, it is possible that panelists did not feel comfortable expressing opinions openly, and procedures such as anonymous votes could be explored. Third, it will be important to consider best-practices for CASCADE outputs, including how recent standardized reporting guidelines for consensus-based decision making [18] could be adapted and optimized for this model. Fourth, although our hybrid meeting format facilitated broad, global participation, it is possible that this structure created uneven engagement and feeling of belongingness across members, consistent with past research [42]. Future work could consider how best to structure meeting locations, including virtual meeting elements, to maximizing panelist engagement and sense of community. Finally, although the panel accomplished its goals with success, like many applied decision-making models, it is unclear which aspects of the CASCADE panel most closely contributed to its success, and how these various “levers” might be adjusted to fine-tune and maximize the inclusiveness, efficiency, and productivity of the approach. Future work may consider experimentally manipulating panel structure and conducting human subjects experiments to determine the “active ingredients” that are most predictive of panel success, with the goal of continuously improving the CASCADE process.

## Conclusions

Within this inaugural pilot, the CASCADE model proved to be an efficient and effective model for moving complex inputs toward tangible, actionable decisions in the context of an ongoing clinical trial. Particular strengths of this model included its high efficiency, centering of community interest-holder input, and integration of strategies to reduce cognitive biases inherent to group-based decision making. Next steps will include determining optimal structure for CASCADE panel meetings – including facilitation, timing, format, and pre-meeting inputs. Future work may also empirically compare efficiency and outputs of CASCADE panels when design elements are intentionally manipulated, producing a stronger understanding of the “active ingredients” most important to this approach. However, at present, the CASCADE model shows promise as a platform for facilitating rigorous and rapid community-centered decision making, potentially narrowing the current practice gap between best-practice community-integration and consensus-building approaches in medical research.

## Data Availability

Data sharing is not applicable to this article as no human subject datasets were generated or analyzed. Data related to Project WellCAST, as well as project pre-registrations relevant to the methods described in this manuscript, are available at https://osf.io/5j8xn/.

## Abbreviations

AI: Artificial Intelligence
CASCADE: Community-Engaged Approach for Scientific Collaborations and Decisions
Project WellCAST: Supporting the Well-Being of Caregivers via Telehealth
